# Laparoscopic surgery for strangulated diaphragmatic hernia after radiofrequency ablation for hepatocellular carcinoma: a case report

**DOI:** 10.1186/s40792-021-01291-8

**Published:** 2021-09-16

**Authors:** Yasuaki Kimura, Daisuke Ishioka, Hidenori Kamiyama, Shingo Tsujinaka, Toshiki Rikiyama

**Affiliations:** 1 Department of Surgery, Chichibu Municipal Hospital, Sakuragi-cho 8-9, Chichibu-City, Saitama 368-0025 Japan; 2 Department of Surgery, Saitama Medical Center, Jichi Medical University, Amanuma-cho 1-847, Omiya-ku, Saitama-City, Saitama 330-8503 Japan

**Keywords:** Diaphragmatic hernia, Strangulated hernia, Radiofrequency ablation, Complication, Laparoscopic surgery

## Abstract

**Background:**

Percutaneous radiofrequency ablation (RFA) is an effective treatment for hepatocellular carcinoma (HCC), but delayed thermal damage can cause diaphragmatic hernia (DH). Surgery is recommended for DH, and open surgery is widely accepted. This report presents a case of laparoscopic surgery for strangulated DH that occurred after RFA.

**Case presentation:**

An 80-year-old woman with a history of hepatitis C-induced liver cirrhosis and HCC was admitted to our institution owing to sudden-onset intense epigastric pain. Twenty-two months earlier, she received RFA treatment for HCC located in segment 6/7. Contrast-enhanced computed tomography revealed herniation of the small intestine into the thoracic cavity, with mesenteric fat haziness. Emergency laparoscopic surgery was performed, and the patient was diagnosed with strangulated DH associated with the prior RFA. The defect was closed using absorbable sutures, and the ischaemic small intestine was resected via mini-laparotomy. The patient was discharged on the 10th postoperative day without complications, and no evidence of DH recurrence 15 months after surgery was noted.

**Conclusions:**

Laparoscopic surgery seems useful and feasible for strangulated DH.

## Background

Extended indication of radiofrequency ablation (RFA) for hepatocellular carcinoma (HCC) treatment is associated with the increased incidence of complications. Diaphragmatic hernia (DH) is a rare late-onset complication of RFA for HCC treatment, requiring emergent surgery, especially in cases of strangulated hernia [1]. Accurate diagnosis of DH is difficult owing to its nonspecific symptoms and is occasionally delayed [1]. Unlike laparoscopic surgery, open surgery is a widely accepted surgical approach for DH [2]. However, a recent report showed that the laparoscopic approach has both diagnostic and therapeutic abilities [3]; moreover, laparoscopic surgery is less invasive than open surgery. In the present case, emergent laparoscopic surgery was useful for both the diagnosis and treatment of strangulated DH after RFA.

## Case presentation

**Fig. 1:**
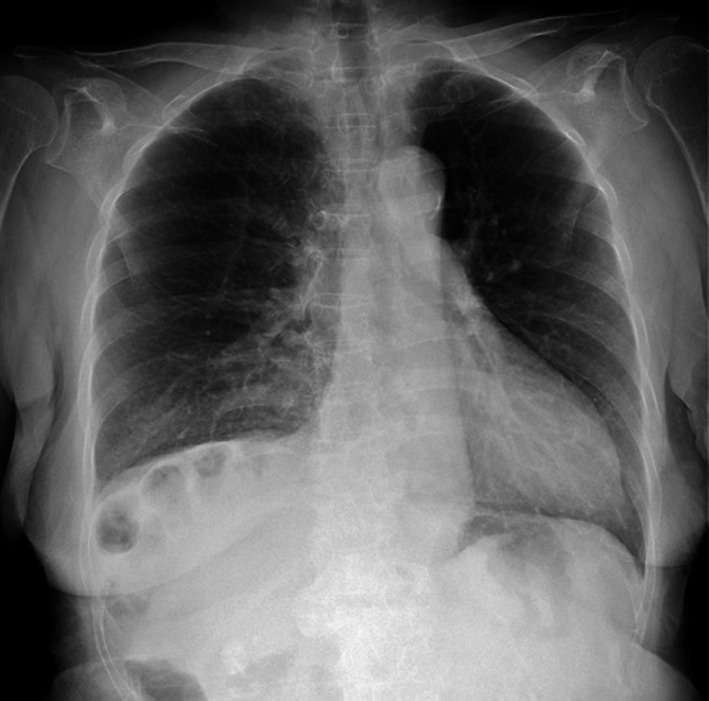
Chest radiography. Chest radiography reveals a massive abnormal intestinal gas above the right lobe of the liver

**Fig. 2:**
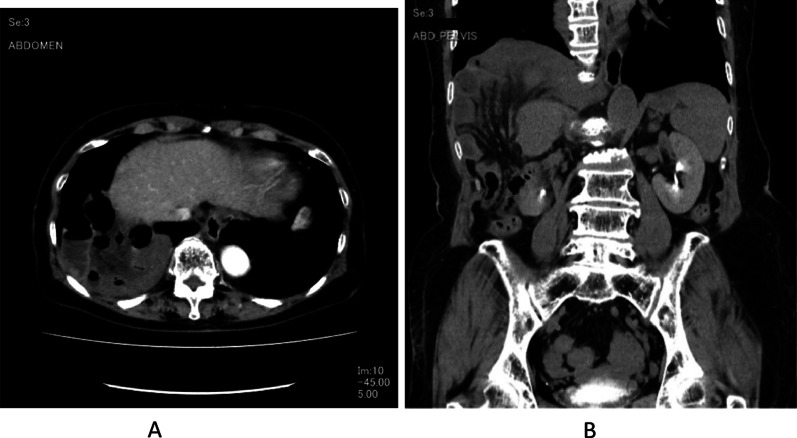
**A** Contrast-enhanced computed tomography (CT). Contrast-enhanced CT showing herniation of the small intestine into the thoracic cavity. **B** Coronal CT image. Coronal CT images showing loops of the small intestine in the thoracic cavity

**Fig. 3:**
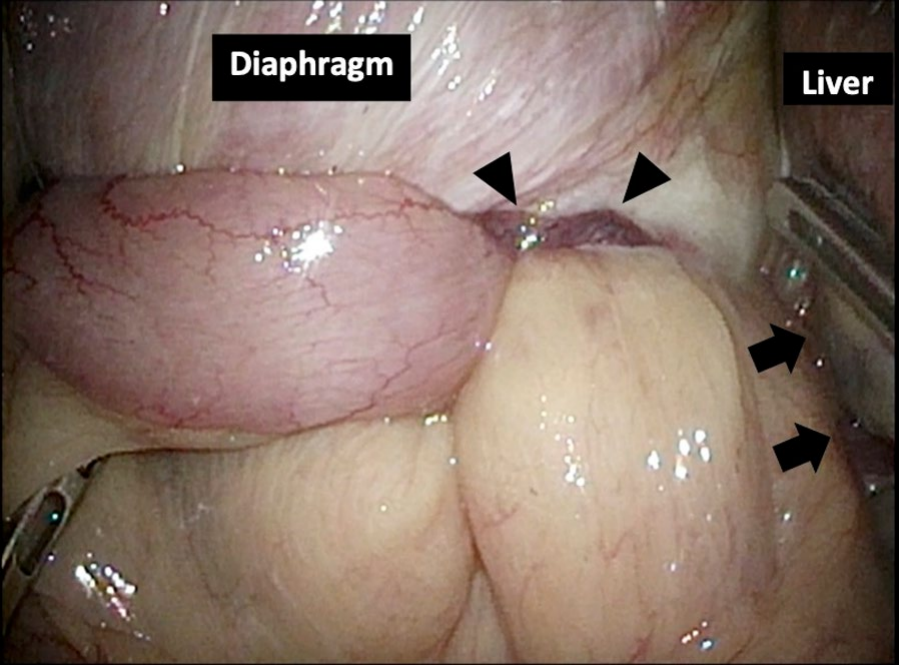
Diaphragmatic hernia (DH). The small intestine is strangulated by the defect in the right side of the diaphragm (arrowheads). The location of the radiofrequency ablation-treated tumour coincides with the site of DH (arrows)

**Fig. 4:**
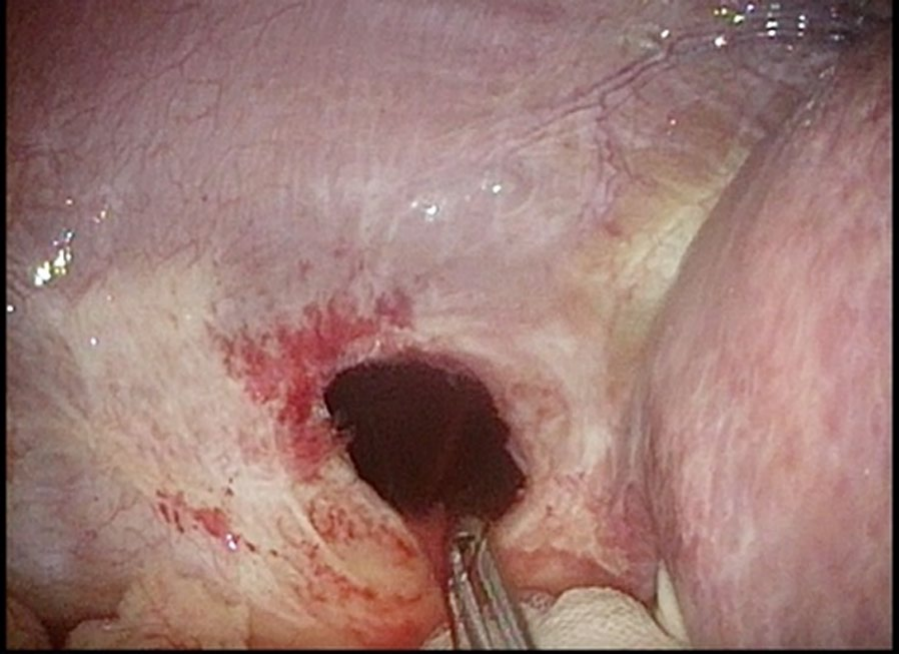
Hernial orifice. The diameter of the hernia defect was approximately 2 cm. The diaphragmatic tissue around the hernial orifice was not fragile by tactile sensation

**Fig. 5:**
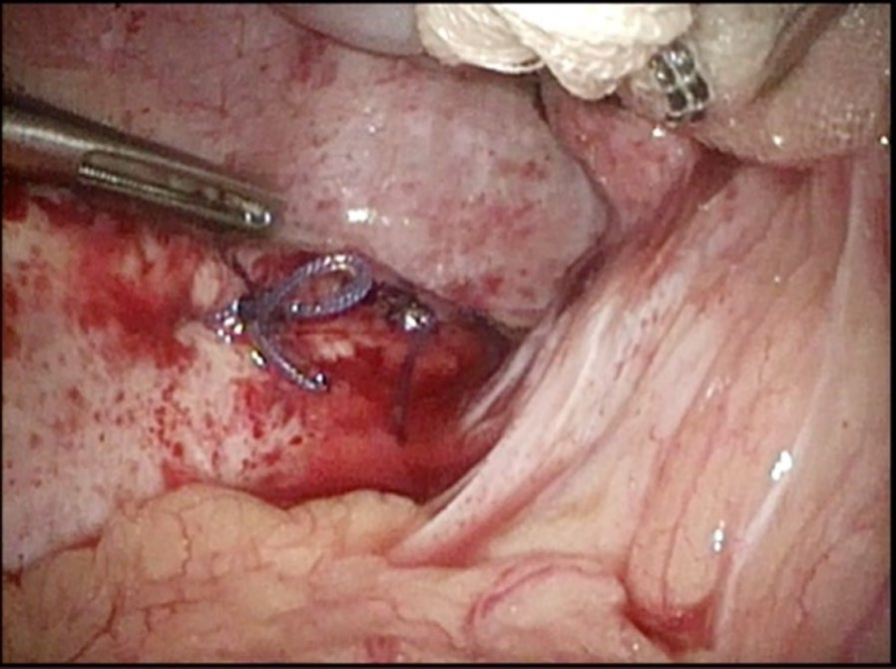
Simple suture repair of the defect

We describe an 80-year-old woman who presented with acute epigastric pain. She had a past medical history of hepatitis C-related liver cirrhosis and HCC in segment 6. She underwent multiple ultrasonography-guided percutaneous RFA sessions using LeVeen Super Slim 3.0 (Boston Scientific, Marlborough, Massachusetts, US). The initial treatment for HCC was performed 25 months previously, without artificial pleural effusion or ascites. The duration of the procedure was 114 min. Three months later, she underwent second treatment for recurrent HCC in segment 6/7 using the identical device with artificial pleural effusion. The duration of the procedure was 125 min. Follow-up computed tomography (CT), which was performed 1 month before admission (21 months after the 2nd RFA treatment), did not show HCC recurrence and DH. No history of trauma was reported. On the day of admission to our institution, she experienced sudden-onset, intense epigastric pain and visited the family doctor. Chest radiography revealed a massive abnormal intestinal gas above the right lobe of the liver (Fig. 1). She was transferred to our hospital for further examination and treatment. Her vital signs, including blood pressure (100/70 mmHg), pulse rate (80/min), and SpO2 (94% at room air), were unremarkable. Physical examination revealed mild tenderness around the umbilicus, with no peritoneal signs. No findings of trauma were noted. Laboratory findings, including white blood cell count and C-reactive protein level, did not demonstrate abnormalities. Regarding hepatic function, she was classified as Child–Pugh Class B, with slight hypoalbuminaemia (3.2 g/dL) and prolonged prothrombin time (63% increase). Contrast-enhanced CT showed small intestine herniation into the thoracic cavity and an atrophic liver. The mesenteric fat of the small intestine was congested; however, no evidence of bowel perforation was noted (Fig. 2A, B). Emergent surgery was performed owing to the suspicion of strangulated DH. In the supine position, the patient underwent laparoscopic surgery under general anaesthesia. A 12-mm port was introduced above the umbilicus using the open Hasson technique. After creating a pneumoperitoneum by carbon dioxide insufflation, we inserted ports in the epigastric region and the left and right flanks. Laparoscopic observation revealed that the small intestine was herniated into the right thoracic cavity through the diaphragmatic defect. The location of the RFA-treated tumour matched the site of the DH (Fig. 3). We gently reduced the contents of the hernia. Pneumothorax occurred, but her haemodynamics and oxygenation were stable. The intruded small intestine showed ischaemic change and partial necrosis, although no fragility was noted. We performed mini-laparotomy and resected the ischaemic small intestine (approximately 100 cm). A chest drain tube was inserted. The diameter of the hernial orifice was approximately 2 cm (Fig. 4). We confirmed by tactile sensation using forceps that the diaphragmatic tissue around the hernial orifice was not fragile. Ascitic fluid accumulated, and abdominal irrigation was performed with a normal saline solution. The defect was completely repaired using 1–0 absorbable braid polyglactin sutures via a continuous suture technique (Fig. 5). The operative time was 161 min, and the blood loss was minimal. The patient was discharged on the 10th postoperative day without complications. Recurrent hernia was not noted in the patient 15 months after surgery.

## Discussion

DH is a rare late-onset complication of RFA for HCC treatment that is potentially life-threatening especially when it results in strangulation [1]. This case report demonstrates that the laparoscopic approach was useful for both the diagnosis and treatment of strangulated DH.

Iatrogenic DH has been reported following surgery, RFA of liver tumours, and excisional biopsy [4, 5]. The incidence of DH after RFA in an institution was reported to be 0.28% (6/2134) over a decade [6]. The main cause of the DH after hepatic RFA is diaphragmatic injury due to mechanical and thermal damage by the RFA needle itself [1, 6]. Mechanical damage by transdiaphragmatic passages of RFA needle may create defects in the diaphragm, leading to chronic DH; thermal damage may cause an inflammatory response, leading to a late-onset defect [1, 6]. It was reported that the incidence of diaphragmatic injury was higher in cases wherein the tumour was adjacent to the diaphragm, patients with large tumours, cases of repetitive RFA, and cases of non-use of artificial ascites [7]. In addition to mechanical and thermal damage, poor liver function might prevent the injured tissue from adequately healing. Furthermore, atrophy of the right lobe of the liver creates space between the diaphragm and the liver, which may contribute to the herniation of abdominal contents and strangulation [1, 6]. The reported interval between hepatic RFA and DH occurrence is 5–24 months (mean 13.3 months) [1]. Initial presentations of DH included abdominal, pulmonary, and obstructive symptoms, which were caused by the herniation of abdominal contents into the thoracic cavity [1]. Severe complications of DH after hepatic RFA were intestinal perforation and empyema secondary to intestinal necrosis, which were reported in four cases [1, 6, 8, 9]. CT is considered the most effective modality for diagnosing DH [10]. Multidetector CT is also useful for identifying anatomical relationships and assessing strangulation [11]. Accurate diagnosis of DH after RFA is made by the coexistence of an hernia defect at the site of RFA without any other obvious causes [11].

Surgical reduction of hernia contents and closure of the defect are recommended immediately following diagnosis or suspicion of DH. Surgery can be performed through transthoracic, transabdominal, and combined thoracoabdominal approaches. Recently, laparoscopic surgery for treating DH after RFA was reported [3]. However, our review of the literature in PubMed, using “diaphragmatic hernia” and “radiofrequency ablation” as keywords, revealed that the laparoscopic approach was used in only three cases [2, 3, 12], and there was no report of the use of the laparoscopic approach for emergency treatment of a similar case of strangulated DH occurring after RFA.

In the present case, the laparoscopic approach provided excellent visualisation and easy access to the diaphragmatic hernial orifice, with a smaller incision than that of open surgery. Laparoscopy was also useful for assessing the pathophysiology of DH and evaluating the viability of the bowel. Laparoscopic hernia reduction and bowel resection via mini-laparotomy were completed without complications, even in the presence of a strangulated DH. The operation was successful owing to the relatively early detection of DH and the localised area of bowel necrosis, as well as the gentle manipulation of the bowel.

The laparoscopic approach was reported to result in reduced morbidity in Bochdalek DH and might facilitate hernia reduction, haemostasis, and adhesiolysis [13]. Moreover, some reports described the feasibility of laparoscopy in similar conditions, such as in strangulated inguinal hernia and strangulated small bowel obstruction [14, 15]. Minimally invasive surgery can be indicated for older adult patients with liver dysfunction and iatrogenic complications, such as in this case.

The laparoscopic approach affects respiratory circulatory dynamics and increases the risk of bowel injury. Pneumothorax is a potential complication of laparoscopic hernia defect repair, and other potential complications include bowel injury during the reduction and handling of the intruded intestine [13]. Blind, forceful traction is discouraged [14]. If irreversible ischaemic changes of the intruded intestine are extensive and the intestine is fragile, laparoscopic hernia reduction can be challenging and dangerous [15]. Rapid conversion to laparotomy is encouraged if there are concerns about complications during laparoscopic hernia reduction. Preemptive conversion, compared to reactive conversion following an intraoperative complication, can reduce postoperative complications by up to 28.6% [16].

DH recurrence after hepatic RFA was reported in a case of elective laparoscopic repair with nonabsorbable sutures [3]. Generally, tension-free repair by nonabsorbable sutures is advocated [17]. Two-layer closure may be useful for maintaining durability; however, no large data favour interrupted over continuous sutures, nonabsorbable over absorbable, or two-layer over single-layer closure [13, 18]. However, mesh repair can avoid recurrence. Recent reports revealed that mesh repair was associated with a lower recurrence rate than suture repair in abdominal wall and hiatus hernia [19, 20]. A recurrence rate of 1.6% was reported in other forms of DH, such as Bochdalek DH, and mesh reinforcement of the repair site was dependent on the defect size (> 8 cm) or the diaphragmatic fragility of the tissues around the hernia defect [13]. Because patients with DH after hepatic RFA commonly present with liver cirrhosis, mesh repair seems useful for avoiding recurrence in these patients. However, cautious mesh fixation seems necessary in areas wherein the diaphragm is relatively thin and is in close proximity to the pericardium [13]. In the present case, it might have been better to perform two-layer closure with nonabsorbable thread retrospectively. Mesh repair was not performed owing to the high risk of surgical site infection related to the partial intestinal necrosis, which required bowel resection, small defect size, and the absence of fragility of tissues around the defect. Other methods to prevent DH recurrence include abdominal or thoracic muscle flaps; however, these methods have the risk for local haemorrhage and residual defects left in the abdominal or thoracic walls [17].

Considering that hernia recurrence is frequently asymptomatic at first, follow-up imaging is needed [17]. In addition, if the recurrence occurs, surgical repair should be performed.

## Conclusion

DH is a critical complication of hepatic RFA; however, its diagnosis is difficult and is occasionally delayed. The possibility of DH should be considered in patients with a prior history of RFA for an HCC adjacent to the diaphragm. Laparoscopic surgery seems useful and feasible for DH, even in cases of strangulation when respiratory circulatory dynamics is stable. However, if there are concerns about complications, preemptive conversion to laparotomy is encouraged. Follow-up imaging should be required after surgery.

## Data Availability

Data sharing is not applicable to this article as no datasets were generated or analysed during the current study.

## Abbreviations

HCC: Hepatocellular carcinoma
RFA: Radiofrequency ablation
DH: Diaphragmatic hernia
CT: Computed tomography
